# Organotypic modelling as a means of investigating epithelial-stromal interactions during tumourigenesis

**DOI:** 10.1186/1755-1536-1-8

**Published:** 2008-12-11

**Authors:** Athina-Myrto Chioni, Richard Grose

**Affiliations:** 1 Queen Mary University of London, Institute of Cancer, Barts and The London School of Medicine and Dentistry, London, EC1M 6BQ, UK

## Abstract

The advent of co-culture approaches has allowed researchers to more accurately model the behaviour of epithelial cells in cell culture studies. The initial work on epidermal modelling allowed the development of reconstituted epidermis, growing keratinocytes on top of fibroblasts seeded in a collagen gel at an air-liquid interface to generate terminally differentiated 'skin equivalents'. In addition to developing *ex vivo *skin sheets for the treatment of burns victims, such cultures have also been used as a means of investigating both the development and repair of the epidermis, in more relevant conditions than simple two-dimensional culture, but without the use of animals. More recently, by varying the cell types used and adjusting the composition of the matrix components, this physiological system can be adapted to allow the study of interactions between tumour cells and their surrounding stroma, particularly with regards to how such interactions regulate invasion. Here we provide a summary of the major themes involved in tumour progression and consider the evolution of the approaches used to study cancer cell behaviour. Finally, we review how organotypic models have facilitated the study of several key pathways in cancer development and invasion, and speculate on the exciting future roles for these models in cancer research.

## Background

Tumourigenesis is a complex process during which tumour cells acquire a sequence of mutations in genes that directly or indirectly control processes such as cell proliferation, survival, migration and invasion. Such mutations may be activating or inactivating and affect proto-oncogenes or tumour suppressor genes, respectively. It is becoming increasingly clear that, despite the accrual of advantageous mutations occurring specifically in the cancer cells, cells in the stroma can play a critical role in mediating tumour growth and progression. Therefore, although simple cell culture studies have given us amazing insight into the cell and molecular biology underpinning cancer cell behaviour, researchers are increasingly turning to more complex and physiologically relevant cell culture models, where more than one cell type is present, to better understand the nature of cancer.

Ultimately, tumour metastasis is the major cause of death for cancer patients. It comprises the formation of secondary tumours by cells escaping from a primary tumour, circulating around the body (via lymph or blood) and becoming lodged at tissue-specific or non-specific sites some distance away [1]. Metastasis involves intimate interactions between cancer cells and their environment at a number of stages [2]. According to the classical 'seed and soil' model of metastasis, the primary tumour is biologically heterogeneous and only some cells gain metastatic ability late in tumourigenesis [3,4]. Furthermore, subpopulations of cells may have a tissue-specific expression profile, predetermining the site of metastasis [5]. However, for the purposes of this review, we have focused on the evolution of techniques to study the relatively early metastatic events. Before turning to these experimental models, we consider the main cell types and behaviours involved in tumour progression.

### Cell adhesion

Adhesive interactions between tumour cells and the surrounding substrata are pivotal to metastatic potential, with decreasing adhesion allowing cancer cells to escape from the primary tumour and acquire a more motile and invasive phenotype [6]. Loss of E-cadherin is a hallmark of epithelial-mesenchymal transition (EMT) and plays a significant role in cancer progression [7]. Furthermore, the transmembrane glycoprotein CD44, that binds principally to hyaluronic acid and chondroitin sulphate in the extracellular matrix (ECM) [8], potentiates cell migration, proliferation and angiogenesis [9]. In addition, CD44 stimulation can rescue cells from apoptosis [10] and induce upregulation of integrins [11].

Integrins are widely implicated in cancer progression [12-14]. These heterodimeric transmembrane receptors provide an essential link between the actin cytoskeleton and ECM during cell migration [15]. They are implicated in all of the main stages of cancer cell progression: penetration of basement membranes; invasion of stromal tissue; intra- and extra-vasation and formation of secondary tumours [16]. Integrins can mediate interactions between cancer cells and a number of ECM components (e.g. laminins, collagens and fibronectin) as well as binding to intercellular adhesion molecules and vascular cell adhesion molecules that are expressed on leukocytes and endothelial cells [17]. Integrin-mediated cell adhesion can trigger a number of signalling cascades, including activation of the Rho family of small GTPases and modulation of the actin cytoskeleton [16,18]. Thus, they facilitate membrane extrusions that are essential for cell spreading on the ECM, where integrins and associated proteins are organised into cell-matrix adhesions in large adhesive multi-protein aggregates [16]. Integrins and adhesion molecules mediating cell-ECM and cell-cell association also play an important role in angiogenesis [19].

### Angiogenesis

For a cancer to metastasise, it must reach the circulation, either via the lymphatic system or via capillaries. The formation of new blood vessels not only provides an exit route for cells into the circulation, it also provides the metabolically active cancer cells with oxygen and nutrients [20]. Without a new blood vessel supply, tumours cannot grow more to than about 1 mm diameter [21,22]. Thus, blocking the angiogenic component of carcinogenesis can produce a small, non-angiogenic tumour that is not lethal, despite the high proliferation rate of cancer cells [23]. Angiogenesis is driven by increased secretion of mitogenic growth factors such as platelet-derived growth factor (PDGF), fibroblast growth factor (FGF) and vascular endothelial growth factor (VEGF) [20] from both cancer and stromal cells [19,24].

### Cell-cell and cell-matrix interactions during invasion

Cell contact and adhesion are considered to be fundamental to metastasis. Invasion occurs initially by cancer cells breaking their links with adjacent epithelial cells, migrating through the ECM and invading blood vessels [14,25]. Matrix metalloproteinases (MMPs), urokinase plasminogen activator/receptor, integrins, cathepsins, cadherins, CD44 and many more specific cell-surface-associated molecules can modulate cell-ECM and cell-cell interactions and thus control cancer cell invasion [4,26]. Tumour cells communicate with the surrounding stromal cells, including macrophages, fibroblasts, endothelial cells and inflammatory cells, as well as between themselves, via soluble growth factors and cytokines, and cell surface proteins, such as cadherins and integrins. Tumour cells releasing chemotactic factors can attract inflammatory cells, thus raising the level of cytokines and growth factors produced by the inflammatory cells [17]. These soluble factors can then act on stromal cells to induce the release of proteases for the degradation of the ECM [17].

### Proteolytic enzymes

Many proteolytic enzymes, including urokinase plasminogen activator, cathepsins B and D, plasminogen and MMPs [17], are involved in matrix degradation and several of these are also of prognostic value in cancer [27]. The engagement of cells with ECM proteins is important for a variety of metastatic cellular processes such as adhesion, proliferation and migration [28] and the secretion of proteolytic enzymes and subsequent ECM proteolysis are recurring events in metastasis. MMPs are generally expressed at moderate levels in normal tissues and their production/activation is increased greatly during tissue remodelling [29]. Their important roles in cancer progression include breaking down local tissue and basement membranes and enhancing tumour-induced angiogenesis, thus allowing tumour invasion and metastasis [30,31]. MMPs can cleave the majority of ECM components and many cell-surface molecules, thus assisting cell migration, cell-cell and cell-matrix interactions and thereby contributing to the formation of a permissive microenviroment [32]. More specifically, MMPs can facilitate the activation of growth factors [33], suppression of tumour cell apoptosis [34], release of angiogenic factors [35] and even enable cancer cells to escape the host immune response [36].

### Immune system

Inflammatory cells such as macrophages and mast cells, which form part of the cancer stroma, not only facilitate the engulfment of apoptotic cells, angiogenesis and proteolytic processes, but can also actively assist other metastatic cell behaviours. There is increasing evidence that direct communication between cancer cells and macrophages results in increased cell migration, invasion and metastasis [37-39]. Macrophages can modulate breast cancer metastasis, with mice lacking colony-stimulating factor-1 (CSF-1), a macrophage-stimulating factor, showing reduced tumour metastasis in a mouse model of breast cancer [40,41]. Interestingly, Condeelis and Pollard [38] state that macrophages in tumours "suppress immune functions and instead adopt trophic roles found during development and repair", consistent with the notion that "tumours are like wounds that do not heal" [22]. Similar to wound healing, macrophages can secrete growth factors (e.g. epidermal growth factor (EGF)) during tumour progression that can alter the behaviour of the tumour cells possibly by providing a chemoattractive signal [42,43]. In fact, EGF produced by macrophages has been shown to be responsible for the recruitment of cancer cells in blood vessels, thus facilitating metastasis [37]. Furthermore, in cutaneous malignancies, mast cells have been shown to be involved in tumourigenesis by suppressing the immune system, facilitating endothelial cell migration and participating in the degradation of the ECM [44].

Taking all of the above into account, it is clear that cancer initiation and progression depend heavily on the active involvement of the stroma. Although originally it was believed that stroma primarily played a structural role, it is now clear that somatic mutations of epithelial cells act in concert with the microenvironment (that is, stroma, which is the supportive platform of the epithelial cells) to drive cancer progression. Stromal components (e.g. fibroblasts or myofibroblasts, endothelial cells, inflammatory cells, adipocytes, smooth muscle cells, nerve cells and the ECM) produce growth factors, cytokines and ECM that orchestrate metastatic cell behaviour via paracrine signals with the epithelial tumour cells [45-48]. Thus, because of the complexity of tumourigenesis, the development of reproducible models that reflect, as accurately as possible, the *in vivo *situation is essential.

### Evolution of models used in tumour biology

Historically, the easiest and most popular way of studying cancer cell behaviour *in vitro *has been two-dimensional (2-D) monolayer culture. Cells isolated directly from a primary source or immortal/transformed cell lines have been grown on plastic or glass surfaces with or without matrix and used in a variety of assays to gain insight into their cell biology. Cell migration has been studied in a number of ways; most simply, a pipette tip is used to generate a scratch wound in a confluent monolayer culture, and wounded cultures subsequently fixed at predetermined timepoints and wound closure imaged using time-lapse microscopy. Pre-treatment of cultures with mitomycin C, to block mitosis, may be used to discern migratory phenotypes from variations in proliferative capacity. Scratch wounding is commonly used to assess the effects of drugs, knock-down or over-expression of genes on the cellular proliferation and/or migration associated with wound closure. Equally, cells may simply be plated at low density and random movements recorded either by time-lapse microscopy or by coating the surface with colloidal gold, such that gold particles are phagocytosed by migrating cells [49]. More complex chemotactic migration can be modelled through the use of chemotaxis chambers, such as the Dunn chamber, where cells are imaged as they migrate up a chemotactic gradient [50]. However, the above assays are all restricted to monitoring migration in two dimensions.

More complex cell migration can be assayed in three dimensions by the use of microporous membranes, first used in developmental signalling studies more than 50 years ago [51]. This approach has resulted in the development of commercially available inserts that can be inserted into cell culture plates to study migration, the most widely used being Transwell^® ^inserts. Cells can be seeded on the top of a such a membrane and left to migrate to the other side through holes (typically 5 or 8 μm for migration/invasion studies) over a defined time period (e.g. overnight), and the number of cells on the underside quantified. Such a system can be used to obtain an objective numerical readout to assess the effects of drugs, or the modulation of target gene expression, on cell migration. Furthermore, by the addition of a matrigel layer on top of the membrane, this assay can be modified to measure invasion, such that cells have to invade though a matrix to reach the underside of the filter [52].

One way to ensure an *in vitro *model is representative of normal tissue architecture is to use organ culture. Organ cultures have been in use since as early as 1897, when Loeb successfully cultured several rabbit organs, including liver, kidney, ovary and thyroid, on small plasma clots in test tubes for up to three days [53]. Organ culture is now used routinely and can be a powerful tool. For example, culturing rings from mouse and rat aortae in three-dimensional (3-D) collagen gels has been used extensively to study angiogenesis [54-56] and many other organs, including prostate [57] and small intestine [58], have also been cultured successfully. Although this approach benefits in that epithelial/endothelial cells are cultured in a relatively physiologically normal microenvironment, the culture period during which the organ remains viable is limited. Furthermore, a major obstacle remains of how to obtain starting material, particularly if the tissue is of human origin.

Animal models are widely used in research and have the advantage that experimental procedures may be carried out in living organisms. Many different diseases, including cancer, have been modelled *in vivo*, and such studies are beyond the scope of this review [59]. The benefits of studying cancer in a whole organism are clear, and the relative ease of genetic manipulation, particularly in mice, coupled with the fact that tumours can develop and progress relatively quickly make animal models extremely powerful. However, there are some disadvantages: although fast tumour development can be considered an advantage, at the same time it may not be a true representation of human tumourigenesis. Furthermore, murine tumours may differ from human both in biological and histological features, particularly regarding hormonal response and metastatic potential [59].

In recent years, 3-D culture models have become increasingly popular in tumour biology. It is clear that cancer is not a homogeneous disease, but an orchestrated interaction between different cell types that is organ and tissue specific. Thus, 3-D culture is becoming the model of choice because it provides a simple and easily manipulated system that shows similar architecture to real tissue and takes into account cell-cell and cell-matrix interactions. Such models are particularly popular in skin studies.

### Organotypic studies in skin pathology

**Figure 1:**
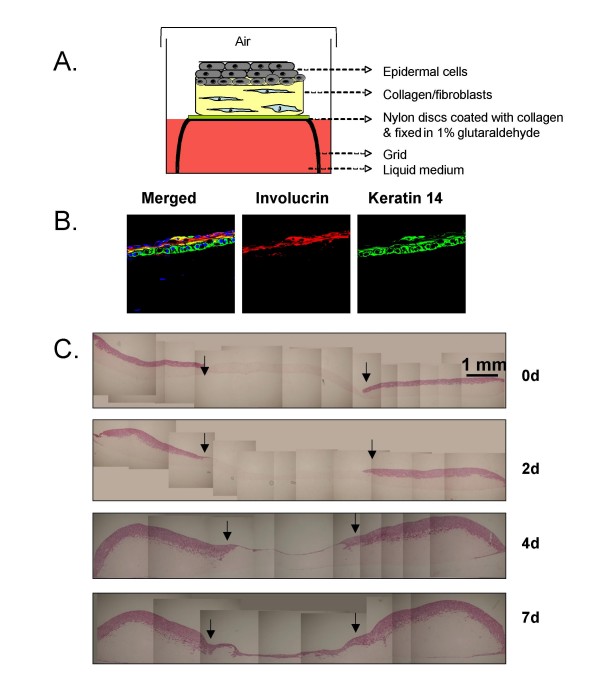
**Organotypic culture of skin equivalent**. **A**. Schematic representation of a skin equivalent organotypic. The stroma consists of collagen and human fibroblasts (5 × 10^5^), with keratinocytes (1 × 10^6^) plated on the top. **B**. Example of immuno-staining for the differentiation markers Keratin14 and Involucrin in a 10 day old culture. **C**. A timecourse of wound healing in an organotypic culture using HaCaT human keratinocytes. A 5 mm wound was created using a punch biopsy and the epidermis (pink) of the organotypic was removed using forceps. Wound closure is shown at different time points (0, 2, 4, 7 days post wound).

Advances in biomedical science have allowed the development of engineered skin tissue substitutes based on data published more than 30 years ago [60-62]. Rheinwald and Green [59] were the first to isolate and culture skin keratinocytes *in vitro*. Bell et al [60] went further and used a collagen layer enriched with dermal fibroblasts as a base on which they seeded keratinocytes (as in Figure 1A), which differentiate as in normal epidermis (as in Figure 1B). In 1981, Burke *et al *[61] used such artificial skin for the successful treatment of an extensive burn injury.

In addition to developing *ex vivo *skin sheets for the treatment of burns victims, such cultures have also been used as a means of investigating both the development and repair of the epidermis, in more relevant conditions than simple 2-D culture, but without the use of animals. Since the early developments in skin engineering, recent publications have focused on the use of skin organotypic co-culture as a model for identifying and investigating the regulatory mechanisms of cell-cell and cell-matrix interactions in skin development that control cell differentiation, tissue homeostasis and tissue integrity [63-66]. All of the above studies have identified the composition and structural organisation of the ECM (i.e. the number of fibroblasts in the dermal equivalent) as one of the most important factors for maintaining normal skin tissue architecture. Interestingly, some studies have been successful in using a combination of cells derived from mouse and human tissues in organotypic skin cultures, allowing the use of genetically engineered mouse cells to analyse specific signalling pathways [64,67]. This was illustrated by elegant studies combining primary human keratinocytes with mouse embryo fibroblasts (MEFs) derived from wild-type, *junB*^-/- ^or *c-jun*^-/- ^mice [68]. This allowed the elucidation of the transcriptional control of an IL-1 dependent pathway whereby IL-1, secreted by keratinocytes, bound to IL-1R on stromal fibroblasts, leading to c-Jun dependent expression and secretion of keratinocyte growth factor (KGF) and granulocyte-macrophage colony-stimulating factor (GM-CSF). These growth factors, in turn, stimulated the proliferation and differentiation of the overlying keratinocytes. Equally, the keratinocyte compartment can be manipulated genetically, as with studies where keratinocytes expressing mutant RAS, and marked with a lineage tracer, were mixed with normal human keratinocytes and allowed to stratify in a 3-D culture prior to treatment with the tumour promoter TPA [69]. This study confirmed the clonal expansion of tumour cells relative to their normal neighbours.

The dermal equivalent in these 3-D cultures can be enriched not only with fibroblasts but with a number of different cell types important in the microenvironment, including myofibroblasts, endothelial cells, inflammatory cells and adipocytes. Recently, human mesenchymal stem cells have been added together with dermal fibroblasts in organotypic skin culture. Wounding studies on these cultures (as in Figure 1C) showed that human mesenchymal stem cells could contribute to wound healing processes but they did not differentiate into keratinocytes [70].

In addition, by varying the cell types used and adjusting the composition of the matrix components, this physiological system can be adapted to allow the study of interactions between tumour cells and their surrounding stroma, particularly with regard to how such interactions regulate cell migration and invasion [71]. The Fusenig lab was the first to use the organotypic skin model to study invasion of squamous cell carcinoma (SCC) cells [72], and such studies have shown that loss of E-cadherin is a key step in SCC progression [73]. Recently, Nystrom et al [73] have developed an objective and quantitative method to analyse SCC cell invasion in organotypic culture. Their data proved the reproducibility of the organotypic culture and also the ability of their quantitative method (the Invasion Index) to measure tumour invasion either in culture or after organotypic gels have been implanted in mice, for up to 6 weeks [74]. Organotypic cultures have also been used to assess cancer invasion in the presence of inhibitors or stimulators of cancer invasion. Pre-treatment of oral SCC cell lines with RNAi (e.g. against HAX-1 or β6 integrin) or membrane-permeable peptides (e.g. to block HAX-1 binding) have been used successfully in organotypic skin cultures [75]. Furthermore, inhibitors (e.g. COX-2 inhibitor) have also been added to cultures to assess their effect on cell invasion [76].

Alongside SCC studies, organotypic cultures have been used widely in understanding melanoma invasion. *In vitro *assays of the metastatic behaviour of melanoma cell lines have yielded contrasting data, dependent on the assay adopted [77]. By admixing melanoma cells together with primary human keratinocytes in a standard skin organotypic model, invasion of melanoma cells follows a pattern closely resembling the clinical scenario [78]. Such cultures represent excellent models with which to screen potential treatments and have been used successfully to analyse the therapeutic potential of oncolytic adenoviruses engineered to target melanocytes [79]. Interestingly, this study also showed the importance of using more than one cell culture approach, with very different treatment efficacy observed in submerged versus air-liquid interface culture conditions. More recently, a similar culture model showed a pro-invasive role for TGF-β, in cultures where primary human melanocytes had been immortalised with SV40 large T antigen and telomerase, prior to further manipulation by PTEN knockdown and mutant Raf expression [80]. Aside from the standard organotypic culture, melanoma cells also have been coated onto microcarrier beads and cultured within a stromal compartment, allowing the development of a novel assay to quantify invasive capacity [81].

### Organotypic cultures in other cancer models

Organotypic cultures have been extended to different tumour types including breast, prostate and ovarian cancer. Similar to the skin approach, human ovarian surface epithelial cells have been grown at an air-liquid interface on collagen gels containing NIH3T3-J2 fibroblasts as feeder cells. Those cultures generated a single layer of flat cells growing on the collagen surface, similar to cells growing *in vivo *[71]. More recently, these 3-D cultures have been used to study stromal progression and stroma-induced ovarian cancer by using fibroblastic cell lines from ovarian tumour samples as well as normal ovarian fibroblasts [82]. In prostate cancer, similar 3-D models have also been used [83]. However, small human prostatic adenocarcinoma fragments also were cultured on collagen sponges for 3 weeks, maintaining the 3-D epithelial and stromal organisation similar to the *in vivo *tumour [84].

Organotypic cultures have also been used extensively in breast cancer research [85-90]. Breast cancer 3-D cultures have been used to investigate gene expression profiles [89,91] and to study the interaction of human epithelial cells with their microenvironment, including fibroblasts, myofibroblasts and ECM [86]. It has been described previously that normal human mammary epithelial cells, when embedded in a 3-D environment, form polarised spheroids with a central lumen similar to the normal mammary gland [92,93]. Importantly, mouse mammary epithelial cells growing in 3-D cultures can respond to lactogenic hormones by producing milk proteins [92,94,95]. The importance of 3-D culture was highlighted by the finding that human epithelial breast cancer cells (MCF-7) showed less sensitivity to anti-estrogen Tamoxifen treatment when cultured in a 3-D scaffold model compared with when grown in monolayer culture [96].

### Future studies

**Figure 2:**
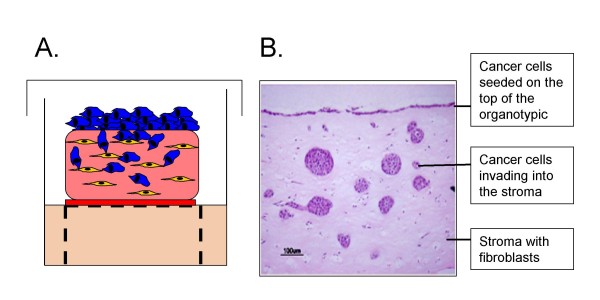
**Breast cancer invasion in an organotypic gel**. **A**. Schematic representation of an organotypic cell culture. The stroma consists of collagen:matrigel (70:30) and 5 × 10^5 ^human fibroblasts (yellow). 1 × 10^6 ^breast cancer cells (blue) are plated on the top of the matrix. The organotypic is then cultures on a grid at the air-liquid interface. **B**. H & E staining of a section through an organotypic culture 9 days after seeding breast cancer cells (MDA-MB-231) on the top. The image shows breast cancer cells remaining on top of the organotypic culture as well as cells invaded into the stroma.

Organotypic cultures have shown that it is possible to recreate, in the laboratory, a histologically similar tissue equivalent for several tissue types, using just two cell types and a matrix. Such relatively basic models are simple to prepare, taking less than 2 weeks to grow, and can be used to study cell migration and invasion with relative ease. For example, skin equivalents can be wounded [97] as illustrated (Figure 1C), generating a model of epithelial repair that is far more realistic than a scratch assay. Cancer cells can be incorporated as illustrated (Figure 2) allowing the assessment of their invasive capacity and, as discussed above, such approaches have already provided valuable information. Future studies will build on these basic principles by increasing the complexity of cell types included in the cultures. Although it is not possible to mimic blood flow in these 3-D cultures, the inclusion of endothelial cells, either together with fibroblasts in the matrix or beneath the matrix, would allow modelling of angiogenesis alongside the study of tumour-stroma interactions. The addition of immune cells to the culture system is another area for development, since numerous studies have highlighted the impact of tumour-associated macrophages on cancer cell biology [98]. Studies where cancer cells have been co-cultured with macrophages on collagen gel have shown clear evidence of intercellular signalling loops that control cell movement [99], and similar experiments are achievable with the organotypic culture approach [100].

One of the challenges facing the field is how to establish live cell imaging in organotypic cultures. Labelling cells fluorescently, either through the use of a dye or by transfection with a fluorescent reporter construct, would facilitate tracking of cell movement by microscopy, but such a technique would be difficult in organotypic cultures grown on grids. More likely, simpler models where cultures can be grown in multi-well format will prove more valuable, as has been shown for breast cancer cells growing as reconstructed organoids, together with fibroblasts, in a collagen gel [101]. Techniques for imaging cancer cells *in vivo *already are proving powerful [102] but, for high-throughput target validation or drug screening assays, *in vivo *studies are neither ethical nor feasible. Understanding the biological relevance of target molecules, particularly using RNAi-based approaches, will be possible not only for the cancer cells themselves but also the stromal cells with which they are associated. This is of great importance given the growing acceptance of the critical role the microenvironment plays in tumour progression. Large-scale functional RNAi screens will be possible, together with more conventional small molecule screening, in the hope that future cell culture studies, based on more physiologically representative models, will be more readily translated into clinically relevant findings.

## Competing interests

The authors declare that they have no competing interests.

## Authors' contributions

A-MC provided the figures and A-MC and RG wrote the manuscript together. Both authors read and approved the final manuscript.
